# Primary Retroperitoneal Echinococcal Cyst Treated With a Total Cystectomy

**DOI:** 10.7759/cureus.53887

**Published:** 2024-02-09

**Authors:** Polyxeni Pichioni, Dimitrios Kokkinovasilis, Stylianos Stylianou, Georgios Kipouridis, Saant Al Mogrampi

**Affiliations:** 1 Department of Surgery, General Hospital of Imathia, Naousa Health Unit, Naousa, GRC

**Keywords:** retroperitneal mass, cystic echinococcosis, giant hydatid cyst, albendazole antihelminthic, total cystectomy

## Abstract

Cystic echinococcosis is a zoonotic disease caused by Echinococcus granulosus and causes significant morbidity, especially in endemic areas. It may remain asymptomatic for a long period. The clinical presentation depends on the exertion of pressure on adjacent organs or the sudden rupture of formed cysts. The presence of primary retroperitoneal echinococcal cysts, with no other organ involvement, has been scarcely reported in the literature. The aim of this study is to present the case of a 69-year-old male complaining of right flank pain for a month. Abdominal CT and MRI were performed, both revealing a large retroperitoneal mass measuring 18 centimeters in diameter, with daughter cysts and spots of calcification. The enzyme-linked immunosorbent assay (ELISA) for hydatid was positive. The patient denied any prior history of hydatidosis; thus, the diagnosis of a primary retroperitoneal echinococcal cyst was established. The patient underwent a successful total cystectomy and is in follow-up, reporting no recurrence of symptoms. Although the presence of echinococcal cysts in locations other than the liver or the lungs is rare, clinicians should always consider the possibility of a hydatid cyst diagnosis and perform the required diagnostic tests.

## Introduction

Echinococcosis is an infection caused by Echinococcus (E.) species, and its clinical course may vary from an asymptomatic infection to severe clinical presentations. The species recognized as causative agents for human echinococcosis include E. granulosus, E. multilocularis, E. vogeli, and E. oligarthrus [1]. Among these species, two are especially important: E. multilocularis, causing alveolar echinococcosis, and E. granulosus, causing cystic echinococcosis. Cystic echinococcosis is endemic in South America, the Mediterranean, Eastern Europe, the Middle East, China, and Russia. In endemic areas, the incidence is estimated from one to 200 per 100,000 people annually [1]. The majority of patients have single-organ involvement, and in approximately 80% of cases, the cysts are localized in the liver and the lungs [2].

The observation of primary retroperitoneal echinococcal cysts without the involvement of other organs is rare and clinically challenging. In this report, we aim to present the case of a 69-year-old male patient with a primary retroperitoneal echinococcal cyst. This case highlights the importance of an accurate preoperative diagnosis of a rare disease in order to apply the required treatment. Our patient was treated successfully with an open total cystectomy followed by the oral administration of antihelminthics.

## Case presentation

We report a case of a 69-year-old male patient complaining of right flank pain starting one month prior. He did not report episodes of diarrhea, fever, or vomiting. There was no jaundice, lymphadenopathy, or obvious palpable mass. The patient mentioned a known history of right carotid artery stenosis to a level less than 50%, for which he had been under medication with acetylsalicylic acid for the last year. The patient had not undergone any surgeries in the past, and his vital signs fell within normal ranges. Upon clinical examination of the abdomen, an umbilical hernia was revealed, while mild discomfort was observed during deep palpation in the right upper and lower abdominal quadrants. Giordano's sign was negative. Laboratory test results were normal and there was no eosinophilia.

**Figure 1:**
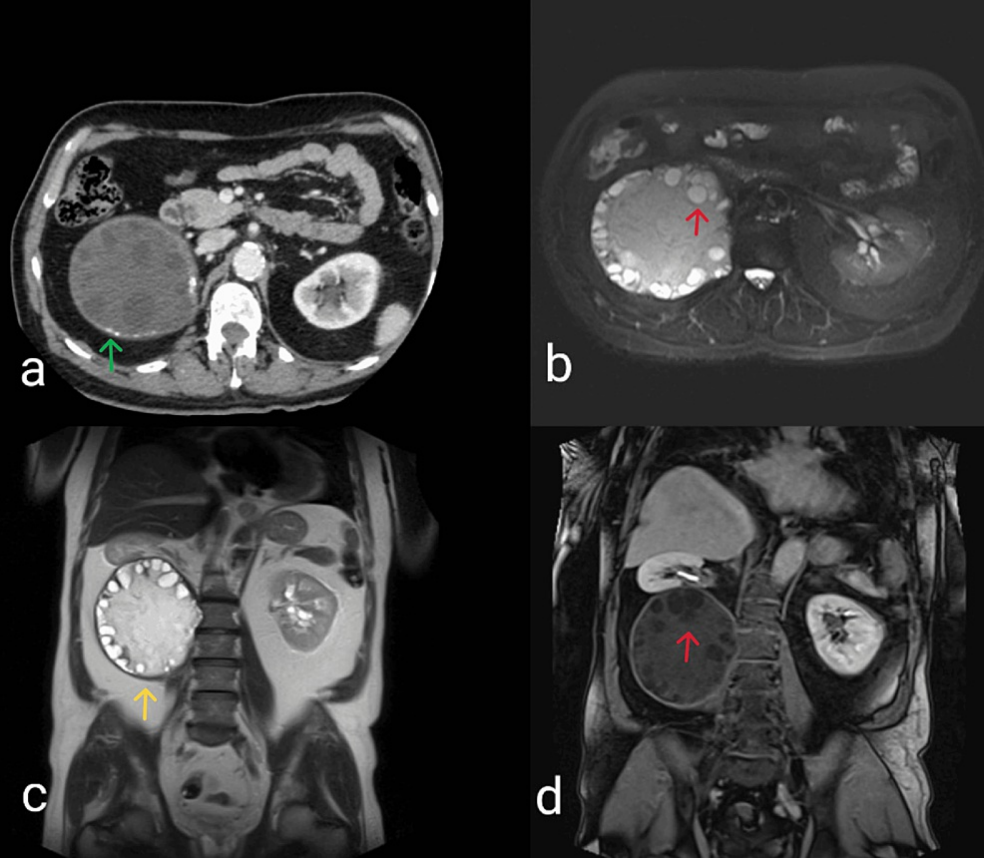
Retroperitoneal hydatid cyst (yellow arrow) with scattered calcifications (green arrow) in the pericyst and daughter cysts (red arrows) lining the periphery of the endocyst. The interior is mostly hydatid sand with a few floating membranes. The cyst is dislocating the right kidney to a completely horizontal position. (a) Contrast-enhanced abdominal CT (axial plane); (b) MRI of the abdomen, T2 weighted image (axial plane); (c) MRI T2 weighted image (coronal plane); (d) T1 fat-saturated MRI sequence (coronal plane).

Subsequently, the patient underwent a contrast-enhanced abdominal CT, which revealed an enormous retroperitoneal mass measuring 18 centimeters in diameter (Figure 1a). The mass was observed to be multiseptated with daughter cysts and random spots of calcification. Pressure from the mass exerted on adjacent organs, especially the right kidney, which was displaced anteriorly to a completely horizontal position. An MRI scan of the abdomen was also performed to establish a firm diagnosis (Figures 1b, 1c, 1d). The findings of the MRI, in accordance with those of the performed CT, confirmed the diagnosis of an isolated retroperitoneal hydatid cyst with daughter cysts and calcification. The ELISA for hydatid was positive. CT scans of the head and chest revealed no abnormal lesions. Preoperatively our patient received albendazole for three months.

Our patient underwent explorative laparotomy. A large retroperitoneal cyst was observed below the right kidney. No other abnormal lesions were detected upon careful examination of the rest of the abdominal cavity. An open total cystectomy was performed. Initially, 95% ethanol was used for the intraoperative killing of protoscoleces and was left within the cyst for 15 minutes. Then, the content of the hydatid cyst was carefully evacuated, and finally, the cyst was completely removed. The histopathology of the mass confirmed the preoperative diagnosis of an echinococcal cyst measuring 18 centimeters in diameter. The postoperative course of the patient was uneventful, and he was discharged on the seventh day post-operation. He was prescribed 400 mg of albendazole twice daily for 12 weeks to reduce the risk of recurrence. He is in follow-up and doing well.

## Discussion

In cystic echinococcosis, the initial infection causes no clinical manifestations in patients, and the small cysts formed remain asymptomatic for a great period of time until they exert pressure on adjacent organs or cause sudden pain due to rupture and spillage of their content [1]. A classification has been developed based on ultrasonography imaging of hydatid cysts to group them into three clinical groups. Cyst types CE1 and CE2 refer to active cysts; the CE3 type represents cysts at a transitional stage, indicating that cysts are starting to degenerate but may also still produce daughter cysts, and finally, the CE4 and CE5 types are inactive cysts that do not contain living protoscoleces [3]. Transitional cysts have been further separated into CE3a and CE3b cysts [4]. The presence of calcification does not reliably suggest the inactive status of cysts; although more often encountered in CE4 and CE5 types, calcification may be detected at all stages [5].

Ultrasonography has traditionally been used as the first imaging method for hydatid cysts. However, if an ultrasound cannot be performed, CT and MRI are viable options. In fact, MRI scans are considered to be a more efficient tool than CT scans when it comes to distinguishing structural features of the cysts [6].

While the presence of hydatid cysts in locations such as the liver and the lungs is anticipated, the observation of such cysts in the retroperitoneum without the involvement of any other organs is very uncommon. It would be wise to include hydatid disease in the differential diagnosis of a retroperitoneal cystic tumor, especially in cases where patients come from endemic areas [7]. The presence of hydatid disease in uncommon sites may be secondary, due to cyst rupture and spillage of its content, or primary through implantation via the lymphatic vessels of the gastrointestinal tract and the hepatic portal system [8]. Secondary retroperitoneal echinococcosis is more common. He et al., in a retrospective study, revealed that only four out of 121 patients diagnosed with retroperitoneal echinococcosis had no prior presence of echinococcal cysts [9].

The first primary retroperitoneal echinococcal cyst was reported in 1958 [10]. Since then, a few similar cases have been reported in the existing literature [7,11-14]. In a retrospective study of 121 cases of retroperitoneal echinococcosis, 68 patients were treated surgically. The authors revealed that 27 of the surgically treated patients had previously undergone surgical resection of liver lesions. Therefore, it has been suggested that, especially in cases involving resection of hydatid cysts in the right liver lobe, the avoidance of cystic content spillage is of utmost importance [9].

As far as cystic echinococcosis's treatment is concerned, the following options exist: surgery, PAIR (puncture, aspiration, injection, and reaspiration), chemotherapy, and the “watch and wait” technique [2]. Each treatment option is chosen based on the structural features and the stage of the cysts, the location, and the patient’s symptomatology and general condition. For echinococcal cysts in extrahepatic locations, therapy varies from the administration of antihelminthic drugs for small asymptomatic cysts to total cystectomy for large symptomatic ones [15]. Surgery, among other indications, is considered suitable as a treatment option for the complete removal of cysts exerting pressure on adjacent organs [2], as in our case, where our patient’s retroperitoneal cyst completely dislocated the right kidney.

The total removal of the cyst is called a pericystectomy, or more accurately, a cystectomy, and is described as closed when the cyst is removed without opening it, and open when protoscolicides are used to kill the metacestodes before the cyst's content is evacuated, and the pericystic tissue is completely removed [2]. When a total cystectomy is not considered a viable treatment option due to adhesions to adjacent organs, then a partial cystectomy should be implemented [11]. It is vital during the procedure to prevent spillage of the cyst's content and to protect the peritoneal cavity through the application of pieces of gauze soaked in 20% hypertonic saline solution [15].

## Conclusions

Retroperitoneal echinococcosis is a rare occurrence, especially when it comes to patients with no prior history of echinococcosis. High clinical suspicion is the key to an accurate diagnosis. A total cystectomy is the gold standard treatment. When it is not possible to excise the hydatid cyst intact, then a partial cystectomy may also be considered. Extreme caution should be taken to avoid spillage of the cyst’s content and contamination.
